# Inter- and Intra-Observer Repeatability of Quantitative Whole-Body, Diffusion-Weighted Imaging (WBDWI) in Metastatic Bone Disease

**DOI:** 10.1371/journal.pone.0153840

**Published:** 2016-04-28

**Authors:** Matthew D. Blackledge, Nina Tunariu, Matthew R. Orton, Anwar R. Padhani, David J. Collins, Martin O. Leach, Dow-Mu Koh

**Affiliations:** 1 CR-UK Cancer Imaging Centre, Radiotherapy and Imaging Division, The Institute of Cancer Research and The Royal Marsden NHS Foundation Trust, London, United Kingdom; 2 Paul Strickland Scanner Centre, Mount Vernon Cancer Centre, Middlesex, United Kingdom; University of Chicago, UNITED STATES

## Abstract

Quantitative whole-body diffusion-weighted MRI (WB-DWI) is now possible using semi-automatic segmentation techniques. The method enables whole-body estimates of global Apparent Diffusion Coefficient (gADC) and total Diffusion Volume (tDV), both of which have demonstrated considerable utility for assessing treatment response in patients with bone metastases from primary prostate and breast cancers. Here we investigate the agreement (inter-observer repeatability) between two radiologists in their definition of Volumes Of Interest (VOIs) and subsequent assessment of tDV and gADC on an exploratory patient cohort of nine. Furthermore, each radiologist was asked to repeat his or her measurements on the same patient data sets one month later to identify the intra-observer repeatability of the technique. Using a Markov Chain Monte Carlo (MCMC) estimation method provided full posterior probabilities of repeatability measures along with maximum a-posteriori values and 95% confidence intervals. Our estimates of the inter-observer Intraclass Correlation Coefficient (ICC_inter_) for log-tDV and median gADC were 1.00 (0.97–1.00) and 0.99 (0.89–0.99) respectively, indicating excellent observer agreement for these metrics. Mean gADC values were found to have ICC_inter_ = 0.97 (0.81–0.99) indicating a slight sensitivity to outliers in the derived distributions of gADC. Of the higher order gADC statistics, skewness was demonstrated to have good inter-user agreement with ICC_inter_ = 0.99 (0.86–1.00), whereas gADC variance and kurtosis performed relatively poorly: 0.89 (0.39–0.97) and 0.96 (0.69–0.99) respectively. Estimates of intra-observer repeatability (ICC_intra_) demonstrated similar results: 0.99 (0.95–1.00) for log-tDV, 0.98 (0.89–0.99) and 0.97 (0.83–0.99) for median and mean gADC respectively, 0.64 (0.25–0.88) for gADC variance, 0.85 (0.57–0.95) for gADC skewness and 0.85 (0.57–0.95) for gADC kurtosis. Further investigation of two anomalous patient cases revealed that a very small proportion of voxels with outlying gADC values lead to instability in higher order gADC statistics. We therefore conclude that estimates of median/mean gADC and tumour volume demonstrate excellent inter- and intra-observer repeatability whilst higher order statistics of gADC should be used with caution when ascribing significance to clinical changes.

## Introduction

Studies conducted using the whole body DWI (WB-DWI) technique have shown high sensitivity for detecting bone marrow and soft tissue diseases, and high diagnostic accuracy for disease staging [1–10]. Due to improved disease/background-tissue contrast on the high b-value images, defining multiple Regions of Interest (ROIs) for tumour analysis throughout the body is possible; a feat that has been enhanced by semi-automatic segmentation techniques [11,12]. These multiple ROIs may be combined to derive quantitative imaging biomarkers that reflect the disease extent by quantifying the tumour *total diffusion volume* (tDV in milliliters), as well as the *global apparent diffusion coefficient* (gADC) (in mm^2^/s), which reflects tissue cellularity [9].

Initial work has demonstrated promising results that the tDV and gADC may be useful for evaluating treatment response in patients with metastatic bone disease, where standard morphological imaging is suboptimal [11]. Clearly, the ability to derive multiple quantitative imaging biomarkers from a single radiological examination is highly attractive. However, current practice requires users to, at best, make use of semi-automatic tools to define and correct ROIs defined in WB-DWI, or rely on manual ROI definition, both of which are associated with errors and bias in the acquired biomarker values. Hence, knowledge of the intra-observer repeatability and inter-observer agreement for tDV and gADC derived from WB-DWI is critical for wider adoption of the technique for disease response evaluation.

The purpose of this study was to determine the inter- and intra-observer variability of two radiologists (R1 and R2) in quantifying WB-DWI parameters (tDV, gADC and associated histogram distribution indices) in a cohort of patients with bone metastases by using a semi-automatic segmentation technique. In this setting we consider whole-body imaging to cover a fields-of-view that includes the neck, chest, abdomen and pelvis.

## Materials and Methods

### Ethics statement

The Royal Marsden Research and Ethics committee approved the study. As this was a retrospective evaluation of prospectively acquired data, signed informed consent was waived. All patient information was de-identified and anonymised prior to analysis.

### Study population

We retrospectively evaluated images of nine consecutive patients with metastatic bone disease who underwent WB-DWI examinations as part of routine clinical care and met our inclusion criteria. Five patients had primary prostate cancer and four patients had primary breast cancer (mean age = 52.4 years, range = 37–70 years). The inclusion criteria were: (1) Patients with predominant metastatic bone disease demonstrated on CT, MRI, skeletal scintigraphy and/ or ^18^FDG-PET, (2) Patients who showed recent disease progression, and were about to commence anti-tumor treatment. Imaging was performed before commencement of treatment.

### MRI technique

Images were acquired at two institutions (five patients at the first and four at the second) using 1.5T MR imaging systems (Avanto, Siemens Healthcare, Erlangen, Germany). Diffusion-weighted MR images were acquired axially during free breathing from the skull vertex to mid-thigh in each patient using the following imaging parameters: repetition time (TR) = 7100-14800ms, echo time (TE) = 65–69.6ms, matrix size = 128x128-150x150, slice thickness = 5mm, receiver bandwidth = 1628–1961 Hz/pixel, 4–7 signal averages, STIR fat suppression with an inversion time (TI) of 180 ms, imaging field of view = 380–430 mm^2^ depending on patient size. All images were acquired using 2 b-values (b = 50, 900 s/mm^2^) for calculation of ADC maps using mono-exponential fitting. The lower b-value of 50 s/mm^2^ was chosen to reduce perfusion effects at the lower b-value and to provide radiologists with ‘black blood’ images [13]. Imaging protocols were optimized to reduce geometric distortions associated with DWI and maintain high SNR, high voxel resolution and uniform fat suppression using phantom and volunteer studies [14].

### Image analysis and processing

Image analysis and processing was performed by two independent radiologists, R1 and R2, with eight and four years’ experience in reading WB-DWI studies respectively using in-house software developed with IDL (Exelis Visual Information Solution, Inc.). Semi-automatic segmentation of disease in each patient was achieved via the following steps (see [11] for further details):

1. Computed DWI (cDWI) [12] was used to visually maximize the contrast in the signal between disease and background tissues as rendered on a maximum intensity projection (MIP) display. A median computed b-value of 1070 s/mm^2^ (range 715–1660 s/mm^2^) was required to obtain optimal visual contrast between disease and background tissues. This was greater than the maximum acquired b-value (900 s/mm^2^).
2. A threshold was manually selected that provided an initial classification of disease from background. A Markov random field prior model for the classification provided smoother segmentation.
3. All segmentation results were visualized as individual regions of interest using a surface rendered display on a MIP and/or multi-planar reformat viewer. Spurious regions of interest (e.g. those outside the patient field-of-view) were manually removed or corrected by the radiologists until they approved of the final disease classification/ segmentation.
4. Resultant regions were also manually corrected using the following exclusion criteria: regions of necrosis with ADC > 2.0 × 10^−3^ mm^2^/s (T2 shine-through), regions that included incomplete fat suppression and any regions above the C4 vertebra to avoid artefacts from susceptibility effects or suboptimal fat suppression.

From the segmentation process described above (further detail provided in [11]), all the regions of interest defined were used to compute the total disease volume (tDV) of metastatic bone disease, which was reported in milliliters (ml), and then transformed via a log function to reduce the scaling effects of errors (log-tDV). By transferring the regions of interest to the ADC map, we also derived summary statistics for whole body gADC histogram analysis. For each patient the following whole-body global ADC (gADC) statistics were calculated: ***gADC-median*, *gADC-mean*, *gADC-variance*, *gADC-skewness*, *gADC-kurtosis*** along with the logarithm of the total diffusion volume, ***log-tDV***.

Each radiologist performed the same analysis on all patients twice separated by one month to minimize recall bias, so that both intra-observer and inter-observer repeatability could be assessed in a joint model.

### Statistical considerations

We applied the following mixed-effects model to our data:

$$Y_{ijk}=\mu +a_{i}+ab_{ij}+\varepsilon_{ijk}$$

- *i* ∈ {1,2,…,*P*} is the patient index
- *j* ∈ {1,2} is the reader identity
- *k* ∈ {1,2} is the k^th^ measurement made by a particular reader on a certain patient
- *Y*_*ijk*_ is the observed WB-DWI metric of interest (e.g. gADC-median or log-tDV).
- *μ* is the true population mean for the metric
- *a*_*i*_ ∼ *N*(0, *σ*_*a*_) denotes the true deviation from σ for the i^th^ patient
- *ab*_*ij*_ ∼ *N*(0, *σ*_*ab*_) is the bias of the j^th^ reader when measuring a WB-DWI metric for the i^th^ patient.
- *ε*_*ijk*_ ∼ *N*(0, *σ*_*εj*_) is a random error made by the reader when making their k^th^ measurement of the metric.

In this article we only describe data from two observers and therefore cannot obtain observer population statistics. This imposes the following model constraint (see Shrout and Fleiss [15] for more details):

$$ab_{i1}+ab_{i2}=0$$

The (2P + 5) unknown parameters in this model are *μ*, *a*_*i*_, *ab*_*i*1_ and the standard deviations *σ*_*a*_, *σ*_*ab*_ and *σ*_*εj*_. By obtaining the best fit for the parameters of this model from our data we obtained estimates of both intra- and inter-reader repeatability simultaneously: **Intra-observer repeatability** is identified as the last of the standard deviation parameters, *σ*_*εj*_, which can be calculated for each reader j, whereas **inter-observer repeatability** is attributed to the standard deviation of the bias terms amongst both readers, *σ*_*ab*_. From these estimates we calculated the Coefficient of Variation, *CoV* = *σ*/*μ*, which may in turn be converted into percentage repeatability: 100% × 1.96 × √2 × *CoV* (p < 0.05, two-tailed test). An important consideration when performing such repeatability studies is an evaluation of how these variance terms compare with the interpatient variability, *σ*_*a*_: If the expected variation in a quantitative metric between patients is small compared to the variability of the measuring process, it will likely be less useful as a biomarker for measuring change in an individual patient. On the other hand, if the variability of a measurement process is relatively small compared with the distribution of observed patient values, it provides evidence for robustness of the metric for detecting treatment effect in an individual patient. For this reason we also reported the intra- and inter-observer Intraclass Correlation Coefficients:

$${\text{ICC}}_{j}^{\text{intra}}=\frac{\sigma_{a}^{2}}{\sigma_{a}^{2}+\sigma_{\varepsilon j}^{2}}$$

$${\text{ICC}}^{\text{inter}}=\frac{\sigma_{a}^{2}}{\sigma_{a}^{2}+\sigma_{ab}^{2}}$$

An ICC^inter^ value tending to 1 in this context indicates good agreement between observers relative to the patient variation, whilst a value tending to 0 represents disagreement. An ICC^intra^ tending to 1 indicates that the repeatability of the j^th^ reader was excellent whereas a value of 0 means that the reader was in general not able to repeat the measurement accurately.

Although classical inference may be used to estimate repeatability and ICC values, for example, by using an ANOVA approach as outlined by Shrout and Fleiss [15], we based our estimation on a Markov-Chain Monte-Carlo (MCMC) approach using Gibbs sampling. This method has the added benefit that it provides a full estimation of the distributions for σ and ICC given the data set. From these distributions we obtained the most probable value for each repeatability metric (mode/peak of the distributions) and, more pertinently, established 95% confidence intervals for these results, a value that is often unquoted in repeatability study literature. Mode values of distributions were found using kernel density estimates of all distributions (search grid size of 1000 using Silverman’s approximation for kernel width and a down-sample of the MCMC train to 10,000 samples). Full implementation for the Gibbs sampler is discussed in S1 Appendix. For comparison, we also calculated Bland-Altman plots [16] for each of the parameters derived by each radiologist. Classical inference of *σ*_*εj*_ was achieved by assuming a zero-mean difference for each WBDWI metric (see [16] for details):

$$\sigma_{\varepsilon j}=\sqrt{\frac{1}{P}\sum_{i=1}^{P}{\left(Y_{ij2}-Y_{ij1}\right)}^{2}}$$

## Results

**Fig 1:**
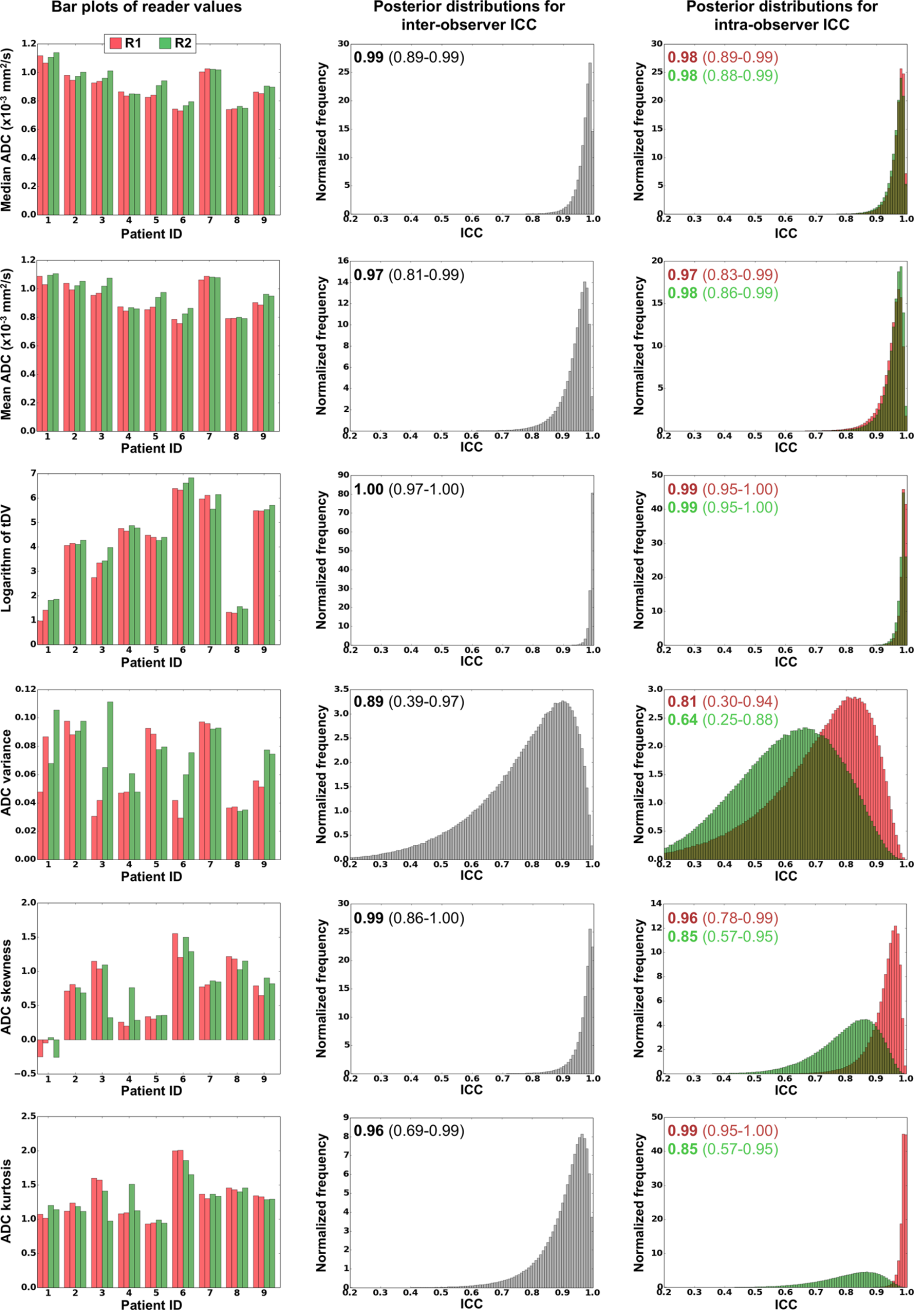
All results for the first radiologist (R1) are displayed in red and those for the second (R2) displayed in green. **Left column**: Bar plots of all parameters estimated in this study. **Centre column**: Posterior distributions for the inter-observer repeatability estimated using MCMC methods. Mode values for the histogram are displayed in bold along with the 95% range for the distribution in parentheses (2.5% - 97.5%). **Right column**: Posterior distributions of the intra-observer ICC values for each parameter. Mode values for the histogram are displayed in bold along with the 95% range for the distributions in parentheses. Note the differences in the intra-observer ICC results for higher-order ADC distribution moments (variance and above). This was largely due to the variation observed in patients 3 and 4 (observed on the bar plots). ADC variance performs especially poorly in these results (ICC = 0.81/0.64), whilst the inter- and intra-observer repeatability is excellent for log-tDV (ICC = 0.99) and mean/median gADC estimates (ICC = 0.97/0.98). R1 also demonstrated good results for gADC skewness and kurtosis parameters (ICC = 0.96/0.99), but this was not the case for R2 (ICC = 0.85).

**Fig 2:**
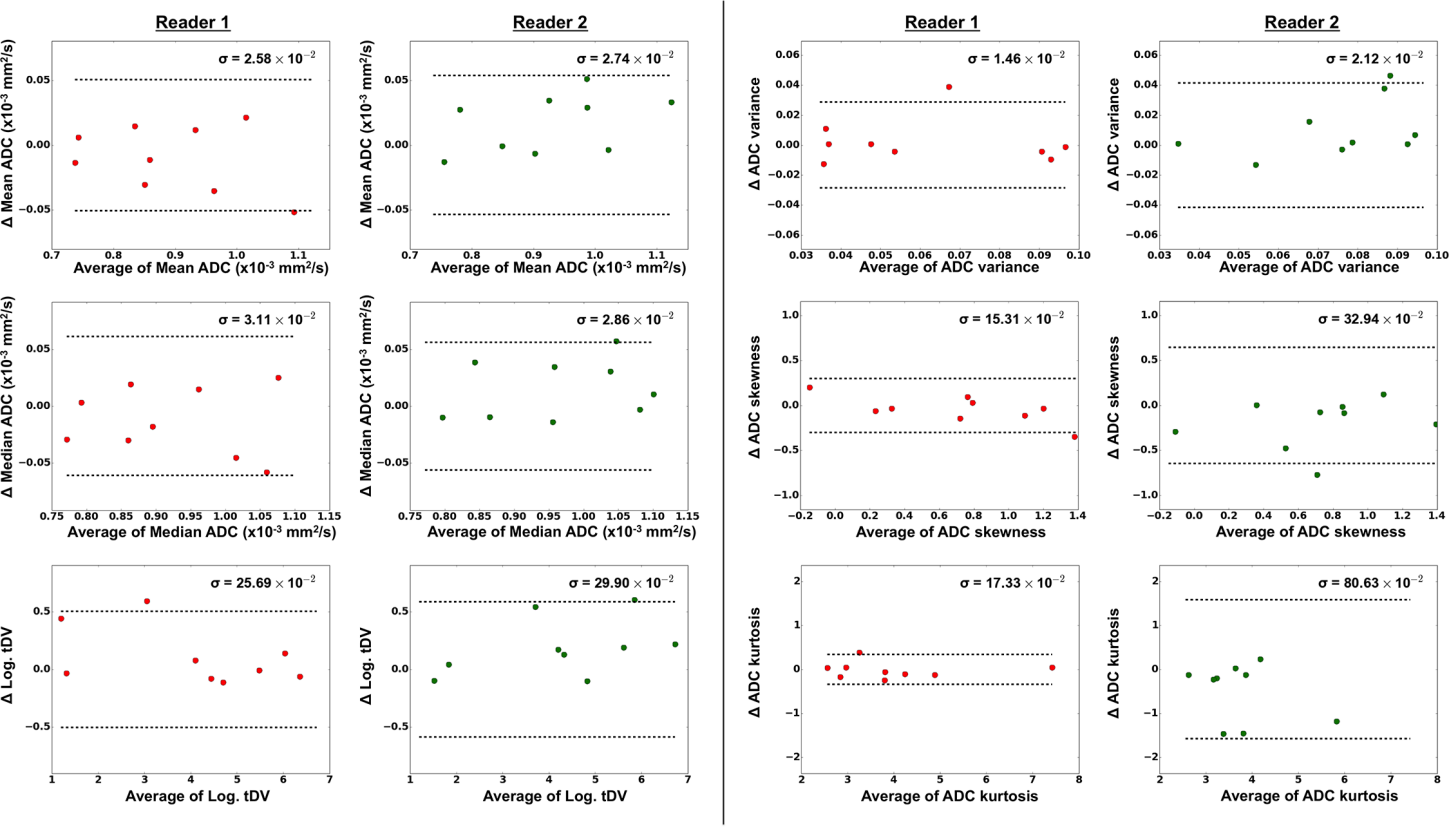
Bland-Altman plots for each parameter of interest, demonstrating the intra-observer repeatability for each of the WBDWI metrics of interest. Results for Reader 1 are plotted on the left in red and those for Reader 2 on the right in green. In all cases there is little evidence of any correlation between differences in repeat estimates of each parameter (vertical axis) and average value (horizontal axis). Estimates of Intra-observer repeatability, σ, are shown on each plot and 95% repeatability intervals (±1.96σ) are represented as dashed horizontal lines.

**Fig 3:**
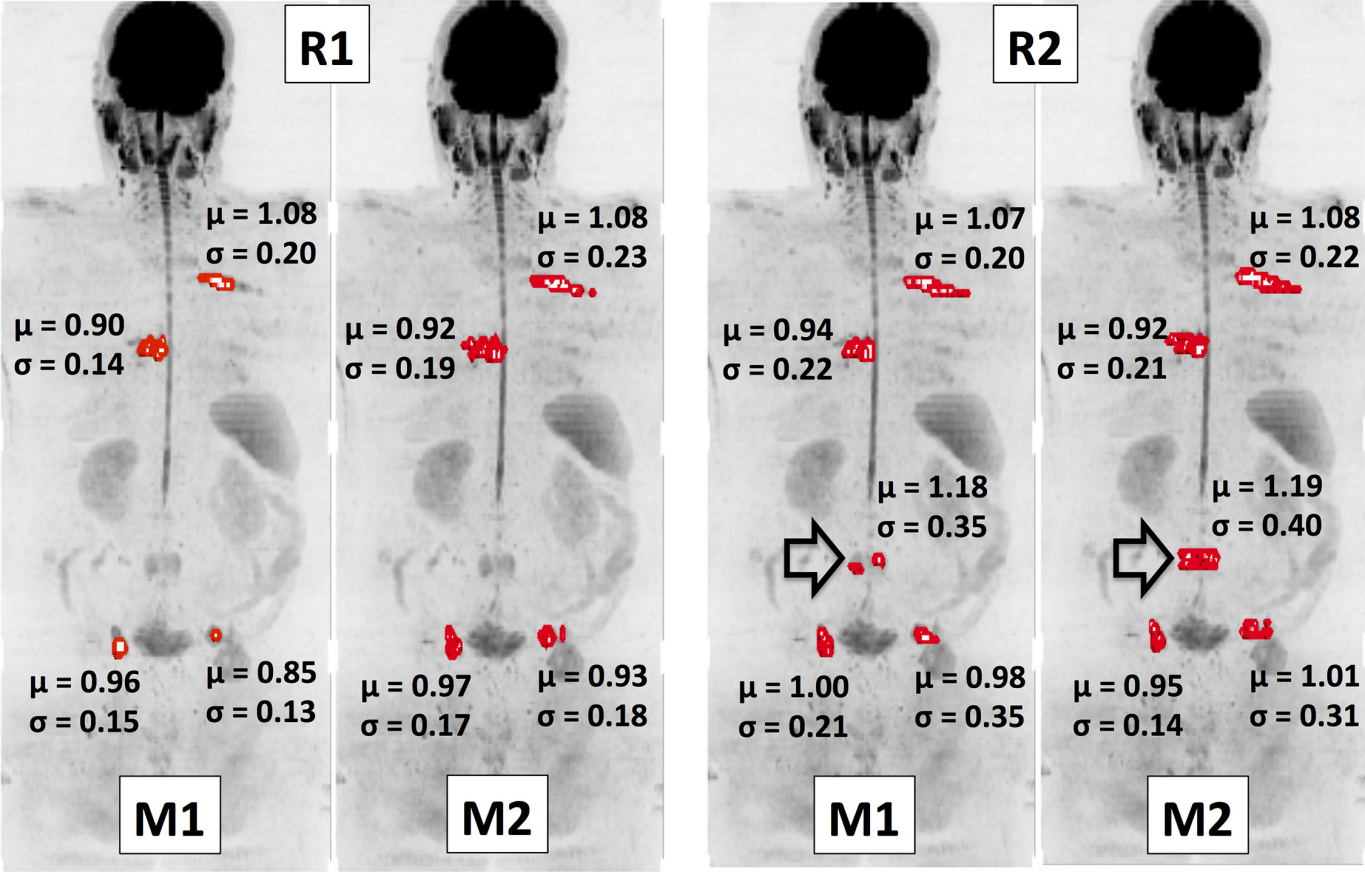
Coronal maximum intensity projection (MIP) of a patient diagnosed with metastatic prostate cancer (b = 900 s/mm^2^ images). Suspected regions of malignancy have been segmented twice (denoted M1 and M2) by each radiologist (denoted R1 and R2) and displayed as red surfaces. The mean ADC value, μ, along with the standard deviation, σ, for each lesion is displayed. It is clear that in general there is good visual agreement between readers of where the disease resides. However, a metastatic site in the lumbar spine (arrow) was not included by R1, as it was thought to represent inactive disease. This disagreement has lead to significantly reduced ICC values in this study demonstrating the sensitivity of high order ADC summary statistics to outliers.

**Fig 4:**
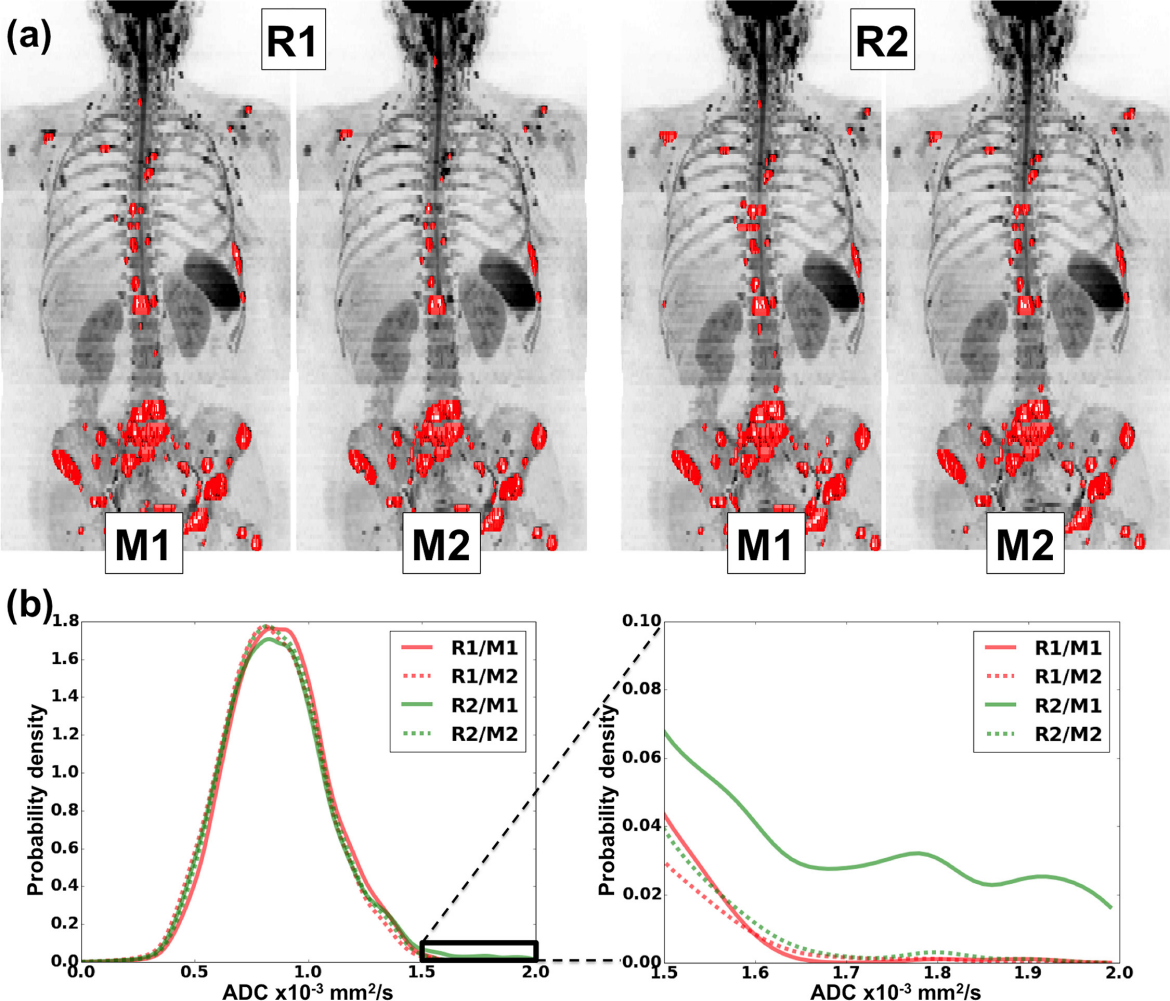
Top row: Coronal maximum intensity projection (MIP) of a patient diagnosed with metastatic breast cancer (b = 900 s/mm^2^ images). Regions of malignancy have been defined twice (denoted M1 and M2) by each radiologist (denoted R1 and R2) and displayed as red surfaces. There is good visual agreement between readers of where the disease resides. **Bottom left**: Kernel density plots of ADC values obtained within regions of interest by each radiologist. The region of the distribution enclosed by the solid black box is demonstrated in more detail in the **bottom right**. The kernel density plots show excellent agreement between the ADC distributions as a whole. However, the presence of a few additional voxels with high ADC in the first measurement by second radiologist (R2/M1) has lead to poor intra-observer repeatability for this radiologist in the higher order ADC statistics of standard deviation, skewness and kurtosis.

Bar-plots for each of the WB-DWI metrics recorded by each observer are displayed in the left column of Fig 1, providing a visual appreciation of the level of discrepancy observed between repeat reading by the same radiologist and the level of agreement between radiologists. The centre column of Fig 1 demonstrates the distributions for estimates of the *inter-observer* ICC for each WB-DWI parameter, whilst the right column provides the distributions for estimates of *intra-observer* ICC. Table 1 summarizes the *inter-observer* repeatability results, whilst Table 2 provides results for the *intra-observer* repeatability of each reader. Fig 2 presents all Bland-Altman plots for each WBDWI-derived parameter of interest, measured by each observer. Classically derived calculations of *intra-observer* repeatability are provided in each case. Figs 3 and 4 provide visual examples of the segmentation results for two of the patient data sets (patient IDs 3 and 4 respectively).

**Table 1:** A summary of the inter-observer repeatability results. All values in bold represent the mode estimate from the estimates distribution of each metric, with 95% confidence intervals displayed in parentheses. All measurements assume ADC units of 10^−3^ mm^2^/s and estimates of *σ*_*ab*_ and **CoV** are displayed following multiplication by a factor 100 for clarity. Note that the percentage repeatability indicates the change in each parameter that would be needed for statistically significant change. Whilst repeatability is excellent for mean/median gADC and log-tDV estimates, poorer reproducibility is found for higher order gADC statistics.

WB-DWI parameter	*σ*_*ab*_ (×100) (95% CI)	CoV (×100) (95% CI)	% Repeatability (95% CI)
**Median gADC**	**1.81** (0.89–3.72)	**1.98** (0.97–4.13)	**5.48** (2.70–11.5)
**Mean gADC**	**2.39** (1.27–4.81)	**2.54** (1.34–5.13)	**7.05** (3.72–14.2)
**log-tDV**	**13.2** (5.99–30.1)	**3.19** (1.40–8.05)	**8.84** (3.88–22.3)
**gADC variance**	**0.95** (0.41–2.02)	**13.6** (5.97–31.1)	**37.8** (16.6–86.1)
**gADC skewness**	**6.32** (2.73–16.7)	**9.09** (3.52–29.8)	**25.1** (9.77–82.7)
**gADC kurtosis**	**33.7** (13.9–71.6)	**8.81** (3.46–19.4)	**24.4** (9.59–53.9)

**Table 2:** A summary of the intra-observer repeatability results. All values in bold represent the mode estimate from the estimates distribution of each metric, with 95% confidence intervals displayed in parentheses. All measurements assume ADC units of 10^−3^ mm^2^/s and estimates of *σ*_*εj*_ and **CoV** are displayed following multiplication by a factor 100 for clarity. Whilst repeatability is excellent for mean/median gADC and log-tDV estimates, we observe a trend for poor reproducibility in higher order gADC statistics. In general, Reader 2 (R2) has worse performance than Reader 1 (R1). R1 demonstrated good reproducibility for gADC kurtosis measurements.

WB-DWI parameter		*σ*_*εj*_ (×100) (95% CI)	CoV (×100) (95% CI)	% Repeatability (95% CI)
**Median gADC**	R1	**1.79** (1.29–3.65)	**1.96** (1.40–4.04)	**5.44** (3.89–11.2)
	R2	**1.90** (1.36–3.83)	**2.10** (1.48–4.27)	**5.82** (4.11–11.8)
**Mean gADC**	R1	**2.16** (1.54–4.42)	**2.32** (1.62–4.69)	**6.43** (4.49–13.0)
	R2	**1.99** (1.42–4.03)	**2.09** (1.49–4.32)	**5.79** (4.13–12.0)
**log-tDV**	R1	**18.2** (12.9–35.6)	**4.29** (2.86–9.61)	**11.9** (7.92–26.6)
	R2	**21.1** (14.8–39.2)	**4.92** (3.28–10.8)	**13.6** (9.08–30.0)
**gADC variance**	R1	**1.05** (0.75–2.27)	**15.7** (10.5–34.2)	**43.6** (29.2–94.9)
	R2	**1.51** (1.05–2.68)	**22.2** (14.5–43.1)	**61.6** (40.2–119.)
**gADC skewness**	R1	**10.8** (7.72–20.7)	**15.2** (9.05–40.2)	**42.2** (25.1–111.)
	R2	**20.9** (15.4–34.3)	**31.3** (17.9–70.1)	**86.6** (49.5–194.)
**gADC kurtosis**	R1	**11.7** (8.54–24.3)	**3.12** (2.08–6.60)	**8.63** (5.75–18.3)
	R2	**54.1** (38.9–98.0)	**13.7** (9.51–26.8)	**37.9** (26.4–74.2)

From Table 1, it is clear that there was excellent inter-observer repeatability in median/mean gADC and also for log-tDV estimates with repeatability of 5.5–9% (median slightly outperforming mean). However, higher order gADC statistics (variance, skewness and kurtosis) all demonstrated poorer inter-observer repeatability of over 20%. The same trend was observed for the intra-observer repeatability results in Table 2 with repeatability values of the order of 5.5–13.5% for mean/median gADC and log-tDV estimates, and more than 30% for higher order gADC statistics (except in the case of kurtosis for R1 who demonstrated approximately 8.5% repeatability). This trend is echoed in the ICC plots displayed in Fig 1 where estimates of inter- and intra-observer ICC for mean/median gADC and log-tDV were greater than 0.97 in all cases, whilst higher order gADC statistics demonstrated lower ICC values due to the poor performance of R2. The best ICC scores obtained were for log-tDV due the larger variation for patient values compared to the mean and median gADC estimates. Classical measurements of intra-observer repeatability *σ*_*εj*_ (Fig 2) were within the ranges expected by MCMC derived values (left column, Table 2). Furthermore, no linear trends were seen in the Bland-Altman plots providing evidence for the Gaussian nature of repeat observer measurements.

Examining two outlier patient cases may elucidate reasons for the reduced performance of higher order gADC statistics. Fig 3 illustrates results for Patient 3, for whom there was significantly large variation in the estimates of gADC variance, skewness and kurtosis of ADC distributions by R2. It is clear from this example that there was disagreement in assessing the disease state in a lumbar vertebra (indicated by an arrow). Although the inclusion of this site by R2 had little impact on mean/median ADC or log-tDV values, the slightly higher ADC of this lesion lead to instability of the higher order ADC statistics. This was again observed in Fig 4, which illustrates detailed results for patient 4. Although this patient demonstrated very low variation in the mean/median ADC and log-tDV, the inclusion of relatively few voxels with high ADC values resulted in unstable higher order ADC statistics. These examples highlight that although ADC histograms may be useful for visual interpretation of ADC changes following therapy, the use of histogram statistical descriptors should be treated with caution, as these may be unstable, particularly in the setting of heterogeneous disease.

## Discussion

Our data show that there is excellent inter- and intra-observer repeatability for estimates of global mean/median apparent diffusion coefficient (gADC) and also for estimates of logarithm of the tumour diffusion volume (log-tDV) derived from whole body DWI in patients with metastatic bone disease. On the other hand, higher order histogram statistics (variance, skewness and kurtosis) derived from gADC measurements demonstrate poorer reproducibility due to their sensitivity to outliers. We therefore recommend prudence when ascribing significance to changes induced by treatments to higher order histogram statistics of gADC distributions, particularly when volumes of interest (VOIs) are prescribed by different observers, or the same observer at different times [11].

Although the inter- and intra-observer repeatability for estimates of log-tDV was poorer compared to mean/median ADC estimates, we believe a high ICC (0.99) indicates that the level of agreement in baseline log-tDV is large enough to warrant use of the segmented tumour volume as a feasible biomarker in WB-DWI studies. Indeed the prognostic value before treatment of tDV is now being investigated in detail with promising results in prostate cancer [17]. Furthermore, we demonstrate that the use of Markov-Chain Monte-Carlo (MCMC) estimation methods are highly attractive for repeatability studies: They provide full descriptions of results including all confidence intervals, especially in the case of ICC measures where confidence intervals can be hard to achieve [15]. We note, however, that this is not the only way to calculate confidence intervals and other methods could be considered [18].

There are limitations to our current study.

1. This was a retrospective study in a small (N = 9), heterogeneous patient population in twin centers (breast & prostate cancer; with patients at various points in their therapies): Future work to define repeatability for individual disease types at the same time points in their therapies could provide additional meaningful information. Nonetheless, we found good inter- and intra-observer repeatability in the mean and median gADC values, which is highly encouraging. Furthermore, our results are in agreement with excellent repeatability found for whole-body ADC estimates in patients diagnosed with multiple myeloma [19].
2. Our current study was confined to evaluating inter-observer and intra-observer errors, and did not incorporate repeated patient measurement to evaluate data acquisition contributions and biological variation induced by therapies. These additional sources of variability will impact adversely on the reproducibility of measurements and their magnitude is currently being evaluated, which would inform the design of multi-centre trials evaluating the prognostic value and therapeutic efficacy prediction of WB-DWI. The impact of these sources of variation has to be understood in the context of observed changes in the median ADC which can be up to approximately 80% in patients responding to treatment [11].
3. Our current tumour segmentation technique is semi-automatic, which can be time consuming, especially when multiple, discontinuous lesions are present, and leads to subjectivity in results. Although fully automatic segmentation strategies for WB-DWI may provide a means of reducing the errors associated with VOI definitions explored in this research, such techniques are still in their infancy and it is expected that expert user-interaction will still be required to delineate disease in patient studies. This will necessitate solid and common training in the use of tools such as ours.
4. Only two radiologists from the same institution were enrolled in this study; a future extension to this work could consider the results from a greater number of clinicians experienced with WBDWI. We expect that this could reduce the range of derived confidence intervals in estimates of inter-observer ICC for each of the parameters evaluated.
5. A multi-center trial, enrolling a larger patient cohort and using scanners from multiple MR manufacturers could modify the confidence intervals in estimates of ICC and related parameters derived in this pilot study.

The emergence of new, targeted therapy has brought optimism for the treatment of metastatic bone disease, which can prolong patient survival and prevent adverse skeletal events [20,21]. However, the inability to identify patients who are not benefitting from such often-costly treatments remains an important clinical challenge. There is thus an urgent need for accurate, quantifiable prognostic and response biomarkers in patients diagnosed with bone metastases. Observer variability makes an important contribution to the repeatability of imaging biomarkers. The current study shows that using WB-DWI, measurement of the disease extent (tDV) and the associated mean or median gADC value has low intra- and inter-observer variability, making it a potential technique for the evaluation of treatment response in bone metastases in the skeleton. It is likely that WB-DWI measurements have the potential to be biomarkers of tumour response in bone metastases.

## Supporting Information

S1 Appendix The implementation of a Gibbs sampler technique for estimation of parameters from a mixed-effects model of observer repeatability. (PDF)
