# Supplementary material for: Inter- and Intra-Observer Repeatability of Quantitative Whole-Body, Diffusion-Weighted Imaging (WBDWI) in Metastatic Bone Disease
Source: PLoS One. 2016 Apr 28;11(4):e0153840. doi: 10.1371/journal.pone.0153840 (PMC4849763; doi:10.1371/journal.pone.0153840)
Supplement: S1 Appendix — (PDF) [file pone.0153840.s001.pdf]

S1 Appendix.

In the following we provide specifications of the steps required in a Gibbs sampler for estimation of a mixed effects model of reader repeatability. Two readers ($j \in \{1, 2\}$) make $k \in \{1, 2, \ldots, M\}$ repeat measurements of some WB-DWI parameter (e.g. gADC or tDV) in $i \in \{1, 2, \ldots, P\}$ patients. Let Y_{ijk} represent the complete set of measurements for a particular WB-DWI parameter: We denote the average over any of the indices with a '$\cdot$' placeholder (for example the matrix of averages across patients is denoted as $\bar{Y}_{\cdot jk}$).

Our data model is

$$Y_{ijk} = \mu + a_i + ab_{ij} + \varepsilon_{ijk} \tag{1}$$

where:

- μ is the population mean for each biological measurement.
- $a_i \sim \mathcal{N}(0, \tau_a^{-1})$ is the deviation from the population mean of the ith patient.
- $ab_{ij} \sim \mathcal{N}(0, \tau_{ab}^{-1})$ gives the interaction effect between reader and patient.
- $\varepsilon_{ijk} \sim \mathcal{N}(0, \tau_{\varepsilon j}^{-1})$ is the error made by the jth reader on their k^{th} measurement of the ith patient.

For simplicity we parameterise the Normal distribution with its precision $\tau = 1/\sigma^2$ (inverse variance) and we impose the constraint $ab_{i1} + ab_{i2} = 0$ due to there being only two readers in this paper (discussed in [1]). By estimating each of the precision terms in 1 given the data we can calculate the intra- and inter-observer Intraclass Correlation Coefficients as follows:

$$\begin{aligned}
\text{ICC}_j^{intra} &= \frac{\tau_a^{-1}}{\tau_a^{-1} + \tau_{\varepsilon_j}^{-1}} \\
\text{ICC}^{inter} &= \frac{\tau_a^{-1}}{\tau_a^{-1} + \tau_{ab}^{-1}}
\end{aligned} \tag{2}$$

Although classical inference may be used to provide ICC values, for example by using an ANOVA approach [1], we use a Markov-Chain Monte-Carlo (MCMC) procedure using Gibbs sampling. This method has the advantage that it provides a sequence of posterior samples for each of the model parameters (τ and ICC) given the data. From these samples we may easily visualise the posterior distribution and determine the maximum a-posteriori (MAP) estimate for each parameter along with descriptors for its error. The full implementation for the Gibbs sampler of model 1 is given below.

S1.1 Gibbs sampling

We wish to obtain an estimate the joint posterior probability density function (*pdf*) of all parameters of interest given the observed data:

$$p(\mu, a_i, ab_{i1}, \tau_a, \tau_{ab}, \tau_{\varepsilon j}|Y_{ijk}) \tag{3}$$

MAP estimation of each parameter may be then achieved by maximising the marginal distribution of 3 for the quantity of interest. However, analytical solutions of the integrals required for marginalisation are typically intractable, and harder yet when MAP estimation of variable transformation (i.e. for ICC estimation) is required. A Gibbs sampler is appropriate for obtaining samples from the full joint *pdf* by constructing a Markov chain that samples each parameter in turn, dependent on the current values of all other parameters. The resulting set of samples for each parameter following the MCMC run is then a vector of samples from the marginal *pdf*, which may be used to infer estimates of MAP and confidence intervals via percentile values (we use 2.5% and 97.5% for $p < 0.05$). Although prior information can be included into this model, we have chosen to use non-informative conjugate priors (normal distributions for μ, a_i and ab_{i1} and gamma distributions for the precision estimates, τ. Pseudocode for obtaining samples from 3 is given below and the conditional distributions required for sampling

at each step are shown in Table 1. Initial values for all parameters ($t = 0$) are obtained using a standard ANOVA approach [1]. In all simulations we drew 10^6 of for each WB-DWI parameter, from which the first 1000 were removed as a burn-in period: All samples thereafter were used for estimation of parameter statistics.

function ICCGibbs(Y_{ijk}, $nMCMC$)

$\mu^0 = \bar{Y}_{\cdots}$

$a_i^0 = \bar{Y}_{i\cdot\cdot} - \bar{Y}_{\cdots}$

$ab_{i1}^0 = \bar{Y}_{i1\cdot} - \bar{Y}_{i\cdot\cdot}$

$ab_{i2}^0 = -ab_{i1}^0$

$\tau_a^0 = (P-1)/\sum_{i=1}^{P}\left(\bar{Y}_{i\cdot\cdot} - \bar{Y}_{\cdots}\right)^2$

$\tau_{ab}^0 = P/\sum_{i=1}^{P}\left(\bar{Y}_{i1\cdot} - \bar{Y}_{i\cdot\cdot}\right)^2$

$\tau_{\varepsilon j}^0 = P/\sum_{i=1}^{P} 2\left(Y_{ij1} - \bar{Y}_{ij\cdot}\right)^2$

for $0 \leq t < nMCMC$ **do**

$\mu^{t+1} \sim p(\mu | a_i^t, ab_{i1}^t, \tau_a^t, \tau_{ab}^t, \tau_{\varepsilon j}^t, Y_{ijk})$

$a_i^{t+1} \sim p(a_i | \mu^{t+1}, ab_{i1}^t, \tau_a^t, \tau_{ab}^t, \tau_{\varepsilon j}^t, Y_{ijk})$

$ab_{i1}^{t+1} \sim p(ab_{i1} | \mu^{t+1}, a_i^{t+1}, \tau_a^t, \tau_{ab}^t, \tau_{\varepsilon j}^t, Y_{ijk})$

$ab_{i2}^{t+1} = -ab_{i1}^{t+1}$

$\tau_a^{t+1} \sim p(\tau_a | \mu^{t+1}, a_i^{t+1}, ab_{i1}^{t+1}, \tau_{ab}^t, \tau_{\varepsilon j}^t, Y_{ijk})$

$\tau_{ab}^{t+1} \sim p(\tau_a | \mu^{t+1}, a_i^{t+1}, ab_{i1}^{t+1}, \tau_a^{t+1}, \tau_{\varepsilon j}^t, Y_{ijk})$

$\tau_{\varepsilon j}^{t+1} \sim p(\tau_{\varepsilon j} | \mu^{t+1}, a_i^{t+1}, ab_{i1}^{t+1}, \tau_a^{t+1}, \tau_{ab}^{t+1}, Y_{ijk})$

$\left(\mathrm{ICC}_j^{intra}\right)^{t+1} = \left(\frac{\tau_a^{-1}}{\tau_a^{-1} + \tau_{\varepsilon_j}^{-1}}\right)^{t+1}$

$\left(\mathrm{ICC}^{inter}\right)^{t+1} = \left(\frac{\tau_a^{-1}}{\tau_a^{-1} + \tau_{ab}^{-1}}\right)^{t+1}$

end for

return $\mu^{1:nMCMC}, a_i^{1:nMCMC}, ab_{i1}^{1:nMCMC}, ab_{i2}^{1:nMCMC}, \tau_a^{1:nMCMC},$
$\tau_{ab}^{1:nMCMC}, \tau_{\varepsilon j}^{1:nMCMC}, \left(\mathrm{ICC}_j^{intra}\right)^{1:nMCMC}, \left(\mathrm{ICC}^{inter}\right)^{1:nMCMC}$

end function

References

[1] P E Shrout and J L Fleiss. Intraclass correlations: uses in assessing rater reliability. *Psychol Bull*, 86(2):420–8, Mar 1979.

Table 1: Summary of update sampling distributions for use in Gibbs sampler of model

Parameter	Conditional distribution
μ	$p(\mu\|\cdot) = \mathcal{N}(\mu', \tau')$ $\mu' = \dfrac{\bar{Y}_{\cdot 1 \cdot}\tau_{\varepsilon 1} + \bar{Y}_{\cdot 2 \cdot}\tau_{\varepsilon 2} - \bar{a}_{\cdot}(\tau_{\varepsilon 1} + \tau_{\varepsilon 2}) - \bar{ab}_{\cdot 1}(\tau_{\varepsilon 1} - \tau_{\varepsilon 2})}{\tau_{\varepsilon 1} + \tau_{\varepsilon 2}}$ $\tau' = PM(\tau_{\varepsilon 1} + \tau_{\varepsilon 2})$
a_i	$p(a_i\|\cdot) = \mathcal{N}(\mu', \tau')$ $\mu' = \dfrac{\bar{Y}_{i1\cdot}\tau_{\varepsilon 1} + \bar{Y}_{i2\cdot}\tau_{\varepsilon 2} - \mu(\tau_{\varepsilon 1} + \tau_{\varepsilon 2}) - ab_{i1}(\tau_{\varepsilon 1} - \tau_{\varepsilon 2})}{\tau_{\varepsilon 1} + \tau_{\varepsilon 2} + \frac{\tau_a}{M}}$ $\tau' = M(\tau_{\varepsilon 1} + \tau_{\varepsilon 2}) + \tau_a$
ab_{i1}	$p(ab_{i1}\|\cdot) = \mathcal{N}(\mu', \tau')$ $\mu' = \dfrac{\bar{Y}_{i1\cdot}\tau_{\varepsilon 1} - \bar{Y}_{i2\cdot}\tau_{\varepsilon 2} - \mu(\tau_{\varepsilon 1} - \tau_{\varepsilon 2}) - a_i(\tau_{\varepsilon 1} - \tau_{\varepsilon 2})}{\tau_{\varepsilon 1} + \tau_{\varepsilon 2} + \frac{\tau_{ab}}{M}}$ $\tau' = M(\tau_{\varepsilon 1} + \tau_{\varepsilon 2}) + \tau_{ab}$
τ_a	$p(\tau_a\|\cdot) = \Gamma(k, \theta)$ $k = \dfrac{P}{2}$ $\theta = \dfrac{2}{\sum_{i=1}^{P} a_i^2}$
τ_{ab}	$p(\tau_{ab}\|\cdot) = \Gamma(k, \theta)$ $k = \dfrac{P}{2}$ $\theta = \dfrac{2}{\sum_{i=1}^{P} ab_{i1}^2}$
$\tau_{\varepsilon j}$	$p(\tau_{\varepsilon j}\|\cdot) = \Gamma(k, \theta)$ $k = \dfrac{PM}{2}$ $\theta = \dfrac{2}{\sum_{i=1}^{P}\sum_{k=1}^{M}(Y_{ijk} - \mu - a_i + (-1)^j ab_{i1})^2}$
